# Supplementary material for: Insights into the Genetic Diversity of Leishmania (Viannia) panamensis in Panama, Inferred via Multilocus Sequence Typing (MLST)
Source: Pathogens. 2023 May 22;12(5):747. doi: 10.3390/pathogens12050747 (PMC10221242; doi:10.3390/pathogens12050747)
Supplement: Supplementary file 1 [file pathogens-12-00747-s001.zip › pathogens-2259471-supplementary/Supplementary Table S1.docx]

**Table S1.** References sequences used in this study to determine *Leishmania* species by an approach based on HSP70 gene.

| **Organism** | **Strain/Isolate** | **GenBanK/TritrypDB Accession** | **Sequence Description** |
| --- | --- | --- | --- |
| *Trypanosoma cruzi* | Sylvio x10/cl1 | CP015689.1 | Chromosome Tcl39 sequence range 473389–475271 |
| *Leishmania panamensis* | MHOM/PA/94/PSC-1 | CP009397.1 | Chromosome 28 sequence range 1065635–1066953 |
| *Leishmania panamensis* | MHOM/COL/81/L13 | AOND01000161 | Whole genome Shotgun sequence range 2903–4866 |
| *Leishmania panamensis* | MHOM/COL/81/L13 | LPAL13_280035900 | Heat Shock Protein 70 gene, putative, complete cds. |
| *Leishmania guyanensis* | MHOM/BR/75/M4177 | EU599093.1 | Heat Shock Protein 70 gene, partial cds. |
| *Leishmania guyanensis* | CDC-204-365 | QVN001000048.1 | Whole Genome Shotgun Sequence range 29057–27739. |
| *Leishmania shawi* | MCEB/BR/1984/M8408 | GU071177.1 | Heat Shock Protein 70 gene, partial cds. |
| *Leishmania braziliensis* | MHOM/BR/M2909 | M87878.1 | Heat Shock Protein 70 mRNA, partial cds |
| *Leishmania braziliensis* | IOC-L3564 | QFBG01000797.1 | Whole Genome Shotgun sequence range 1828–3146 |
| *Leishmania peruviana* | MHOM/PE/03/LH2864 | FN395044.1 | Partial HSP70 gene |
| *Leishmania naiffi* | MDAS/BR/78/M5210 | FN395056.2 | Partial HSP70 gene |
| *Leishmania naiffi* | MDAS/BR/79/M5533 | FR872767.1 | Partial HSP70 gene |
| *Leishmania lainsoni* | CDC-216-34 | RAIA01000124.1 | Whole Genome Shotgun Sequence range 110702–112020 |
| *Leishmania lainsoni* | MHOM/BO/95/CUM71 | FN395047.1 | Partial HSP70 gene |
| *Leishmania mexicana* | MHOM/GI/2001/U1103 | XM_003877072.1 | Putative heat Shock Protein 70, partial mRNA. |
| *Leishmania mexicana* | CDC-215-L49 | RZOC01000008.1 | Whole Genome Shotgun Sequence range 93622–94940 |
| *Leishmania amazonensis* | MHOM/BR/71973/M2269 | LAMA_000567600 | HSP70 gene putative |
| *Leishmania amazonensis* | CDC-210-660 | RZOD01000059.1 | Whole Genome Shotgun Sequence range 1050347 –1051665 |
| *Leishmania donovani* |  | CAAAGX010000028.1 | Genome Assembly contig LdHU3.28, Whole genome Shotgun sequence |
| *Leishmania donovani* | LdCL | CPO29527.1 | Chromosome 28, complete sequence, range retrieved 1065007–1066325 |
| *Leishmania donovani* | CL-SL | LdCL_280035800 | Heat Shock protein 70, putative. |
| *Leishmania donovani* | BPK282A1 | FR799615.2 | Chromosome 28 complete sequence, range retrieved 1092864-1091546. |
| *Leishmania chagasi* | MCER/BR/1981/M6445/Salvaterra | JAGRQE010006929.1 | Whole Genome Shotgun Sequence, segment retrieved 490-1808. |
| *Leishmania infantum* | JPCM5 | UINB01000028.1 | Whole Genome Shotgun Sequence, Genome Assembly contig LinJ28, segment retrieved 1080778-1082096 |
| *Leishmania major* | Friedlin | OU755562.1 | Genome Assembly, Chromosome 28, fragment retrieved 1057691-1059009 |
| *Leishmania major* | SD 75.1 | AFZ101000507.1 | Whole Genome Shotgun Sequence, Genome Assembly contig 28.16, segment retrieved 639-1957 |
| *Leishmania major* | LV39C5 | AODR01000334.1 | Whole Genome Shotgun sequence, fragment retrieved 1129-2389 |
| *Leishmania aethiopica* | CDC_L1204 | RZOE01000006.1 | Whole Genome Shotgun Sequence, fragment retrieved 90954-92214 |
| *Leishmania tarentolae* | Parrot TarII | BLBS01000039.1 | Whole Genome Sequence, Chromosome 28, Draft genome sequence, segment retrieved 1047418-1049390 |
| *Leishmania enriettii* | LEM3045 | ATAF02000376.1 | Whole Genome Shotgun Sequence, segment retrieved 57303-59266 |
| *Leishmania enriettii* | CUR178 | JAFHKP010000028.1 | Whole Genome Shotgun Sequence, Chromosome 28, segment retrieved 1080975-1082938 |
| *Leishmania orientalis* | LSCM4 | JAFHLR010000028.1 | Whole Genome Shotgun Sequence, Chromosome 28, fragment retrieved 3751-5714 |
| *Leishmania martiniquensis* | LSCM1 | JAFEUZ010000028.1 | Whole Genome Shotgun Sequence, Chromosome 28, segment retrieved 1064158-1066121 |
| *Leishmania tropica* | L590 | ATAT01000280.1 | Whole Genome Shotgun sequence, segment retrieved 111461-112770 |
| *Leishmania turanica* | LEM563 | ATBU01000334.1 | Whole Genome Shotgun Sequence, fragment retrieved 28075-29390 |
