# Supplementary material for: Insights into the Genetic Diversity of Leishmania (Viannia) panamensis in Panama, Inferred via Multilocus Sequence Typing (MLST)
Source: Pathogens. 2023 May 22;12(5):747. doi: 10.3390/pathogens12050747 (PMC10221242; doi:10.3390/pathogens12050747)
Supplement: Supplementary file 1 [file pathogens-12-00747-s001.zip › pathogens-2259471-supplementary/Supplementary Table S3.docx]

**Table S3.** Haplotype resolution of the *Leishmania panamensis* isolates found in this study.

| Isolate | Aconitase | ALAT | GPI | HSP70 | DST | Geographical Origin |
| --- | --- | --- | --- | --- | --- | --- |
| FID16203-010 | 1/1 | 1/1 | 1/1 | 1/1 | 1 | Coclé (W) |
| FID16203-028 | 1/1 | 1/1 | 1/1 | 1/1 | 1 | Darién (E) |
| FID16203-034 | 1/1 | 1/1 | 1/1 | 1/1 | 1 | Panamá Oeste (W) |
| FID16203-037 | 1/1 | 1/1 | 1/2 | 1/1 | 2 | Panamá Norte (E) |
| FID16203-038 | 1/1 | 1/1 | 1/1 | 1/1 | 1 | Darién (E) |
| FID16203-042 | 1/1 | 1/1 | 1/1 | 1/1 | 1 | Panamá Este (E) |
| FID16203-044 | 1/1 | 1/1 | 1/1 | 1/1 | 1 | Colón (E) |
| FID16203-046 | 1/1 | 2/1 | 1/1 | 1/1 | 3 | Darién (E) |
| FID16203-050 | 1/1 | 1/1 | 1/1 | 1/1 | 1 | Darién (E) |
| FID16203-052 | 1/1 | 1/1 | 1/1 | 1/1 | 1 | Colón (E) |
| FID16203-054 | 1/1 | 1/1 | 1/1 | 1/1 | 1 | Colón (E) |
| FID16203-055 | 1/1 | 1/1 | 1/1 | 1/1 | 1 | Panamá Este (E) |
| FID16203-062 | 1/1 | 1/1 | 3/3 | 1/1 | 4 | Panamá Norte (E) |
| FID16203-065 | 2/1 | 1/1 | 1/1 | 1/1 | 5 | Bocas del Toro (W) |
| FID16203-069 | 3/2 | 1/1 | 1/1 | 1/1 | 6 | Panamá Oeste (W) |
| FID16203-093 | 3/1 | 1/1 | 1/1 | 1/1 | 7 | Panamá Este (E) |
| FID16203-097 | 1/1 | 1/1 | 1/1 | 1/1 | 1 | Colón (E) |
| FID16203-101 | 1/1 | 1/1 | 1/1 | 1/1 | 1 | Coclé (W) |
| FID16203-104 | 1/1 | 1/1 | 1/1 | 1/1 | 1 | Panamá Oeste (W) |
| FID16203-110 | 1/1 | 2/1 | 1/1 | 1/1 | 3 | Darién (E) |
| FID16203-114 | 1/1 | 1/1 | 1/1 | 1/1 | 1 | Panamá Este (E) |
| FID16203-118 | 1/1 | 1/1 | 1/1 | 1/1 | 1 | Panamá Oeste (W) |
| FID16203-126 | 1/1 | 1/1 | 1/1 | 1/1 | 1 | Panamá Este (E) |
| FID16203-127 | 1/1 | 1/1 | 1/1 | 1/1 | 1 | Colón (E) |
| FID16203-129 | 1/1 | 1/1 | 1/1 | 1/1 | 1 | Panamá Oeste (W) |
| FID16203-139 | 1/1 | 1/1 | 1/1 | 1/1 | 1 | Panamá Oeste (W) |
| FID16203-140 | 3/3 | 1/1 | 1/1 | 1/1 | 6 | Panamá Oeste (W) |
| FID16203-148 | 1/1 | 1/1 | 4/5 | 1/1 | 8 | Panamá Oeste (W) |
| FID16203-166 | 1/1 | 1/1 | 1/1 | 1/1 | 1 | Panamá Este (E) |
| FID16203-170 | 1/1 | 1/1 | 1/1 | 1/1 | 1 | Colón (E) |
| FID16203-174 | 1/1 | 1/1 | 1/1 | 1/1 | 1 | Colón (E) |
| FID16203-176 | 4/4 | 1/1 | 1/1 | 1/1 | 9 | Panamá Este (E) |
| FID16203-178 | 1/1 | 1/1 | 1/1 | 1/1 | 1 | Colón (E) |
| FID16203-179 | 1/1 | 1/1 | 1/1 | 1/1 | 1 | Panamá Este (E) |
| FID16203-182 | 1/1 | 1/1 | 1/1 | 1/1 | 1 | Colón (E) |
| FID16203-189 | 1/1 | 1/1 | 1/1 | 1/1 | 1 | Colón (E) |
| FID16203-227 | 1/1 | 1/1 | 1/1 | 1/1 | 1 | Panamá Oeste (W) |
| FID16203-232 | 3/3 | 1/1 | 1/1 | 1/1 | 6 | Panamá Este (E) |
| FID16203-241 | 1/1 | 1/1 | 1/1 | 1/1 | 1 | Panamá Oeste (W) |
| FID16203-249 | 1/1 | 1/1 | 1/1 | 1/1 | 1 | Panamá Oeste (W) |
| FID16203-256 | 3/1 | 1/1 | 1/1 | 1/1 | 7 | Panamá Oeste (W) |
| FID16203-265 | 1/1 | 1/1 | 1/1 | 1/1 | 1 | Darién (E) |
| FID16203-364 | 1/1 | 1/1 | 1/1 | 1/1 | 1 | Coclé (W) |
| FID16203-413 | 3/1 | 1/1 | 1/1 | 1/1 | 7 | Coclé (W) |
| FID16203-440 | 3/3 | 1/1 | 1/1 | 1/1 | 6 | Coclé (W) |
| FID16203-446 | 1/1 | 1/1 | 1/1 | 1/1 | 1 | Coclé (W) |
| FID16203-453 | 3/1 | 3/3 | 1/1 | 1/1 | 10 | Coclé (W) |
| FID16203-454 | 3/3 | 1/1 | 6/6 | 2/1 | 11 | Coclé (W) |
| FID16203-475 | 3/3 | 1/1 | 1/1 | 1/1 | 6 | Coclé (W) |
| FID16203-479 | 1/1 | 1/1 | 1/1 | 1/1 | 1 | Coclé (W) |
| FID16203-509 | 1/1 | 1/1 | 1/1 | 1/1 | 1 | Coclé (W) |
| FID16203-514 | 1/1 | 1/1 | 1/1 | 1/1 | 1 | Coclé (W) |
| FID16203-529 | 1/1 | 1/1 | 1/1 | 1/1 | 1 | Coclé (W) |
| FID16203-674 | 1/1 | 1/1 | 1/1 | 1/3 | 12 | Bocas del Toro (W) |
| FID16203-684 | 1/1 | 1/1 | 1/1 | 1/1 | 1 | Bocas del Toro (W) |
| FID16203-687 | 1/1 | 1/1 | 1/1 | 1/1 | 1 | Bocas del Toro (W) |
| FID16203-693 | 1/1 | 1/1 | 1/1 | 1/1 | 1 | Bocas del Toro (W) |
| FID16203-695 | 5/5 | 1/1 | 1/1 | 1/1 | 13 | Bocas del Toro (W) |
| FID16203-712 | 1/1 | 1/1 | 1/1 | 1/1 | 1 | Bocas del Toro (W) |
| FID16203-C1404 | 1/1 | 1/1 | 1/1 | 1/1 | 1 | Panamá Oeste (W) |
| FID16203-C14131 | 1/1 | 1/1 | 1/1 | 1/1 | 1 | Panamá Este (E) |
| FID16203-C1602 | 1/1 | 1/1 | 1/1 | 1/1 | 1 | Colón (E) |
| FID16203-C1605 | 1/1 | 1/1 | 1/1 | 1/1 | 1 | Darién (E) |
| FID16203-C1611 | 1/1 | 1/1 | 1/1 | 1/1 | 1 | Panamá Este (E) |
| FID16203-C1625 | 1/1 | 1/1 | 1/1 | 1/1 | 1 | Panamá Este (E) |
| FID16203-C16120 | 1/1 | 1/1 | 1/1 | 1/1 | 1 | Panamá Este (E) |
| FID16203-C1721 | 1/1 | 1/1 | 1/1 | 1/1 | 1 | Panamá Este (E) |
| FID16203-C1819 | 3/3 | 1/1 | 1/1 | 1/1 | 6 | Panamá Oeste (W) |
| FID16203-C1877 | 1/1 | 1/1 | 1/1 | 1/1 | 1 | Colón (E) |

E: eastern Panama; W: western Panama.
