# Supplementary figures and images for: Insights into the Genetic Diversity of Leishmania (Viannia) panamensis in Panama, Inferred via Multilocus Sequence Typing (MLST)
Source: Pathogens. 2023 May 22;12(5):747. doi: 10.3390/pathogens12050747 (PMC10221242; doi:10.3390/pathogens12050747)

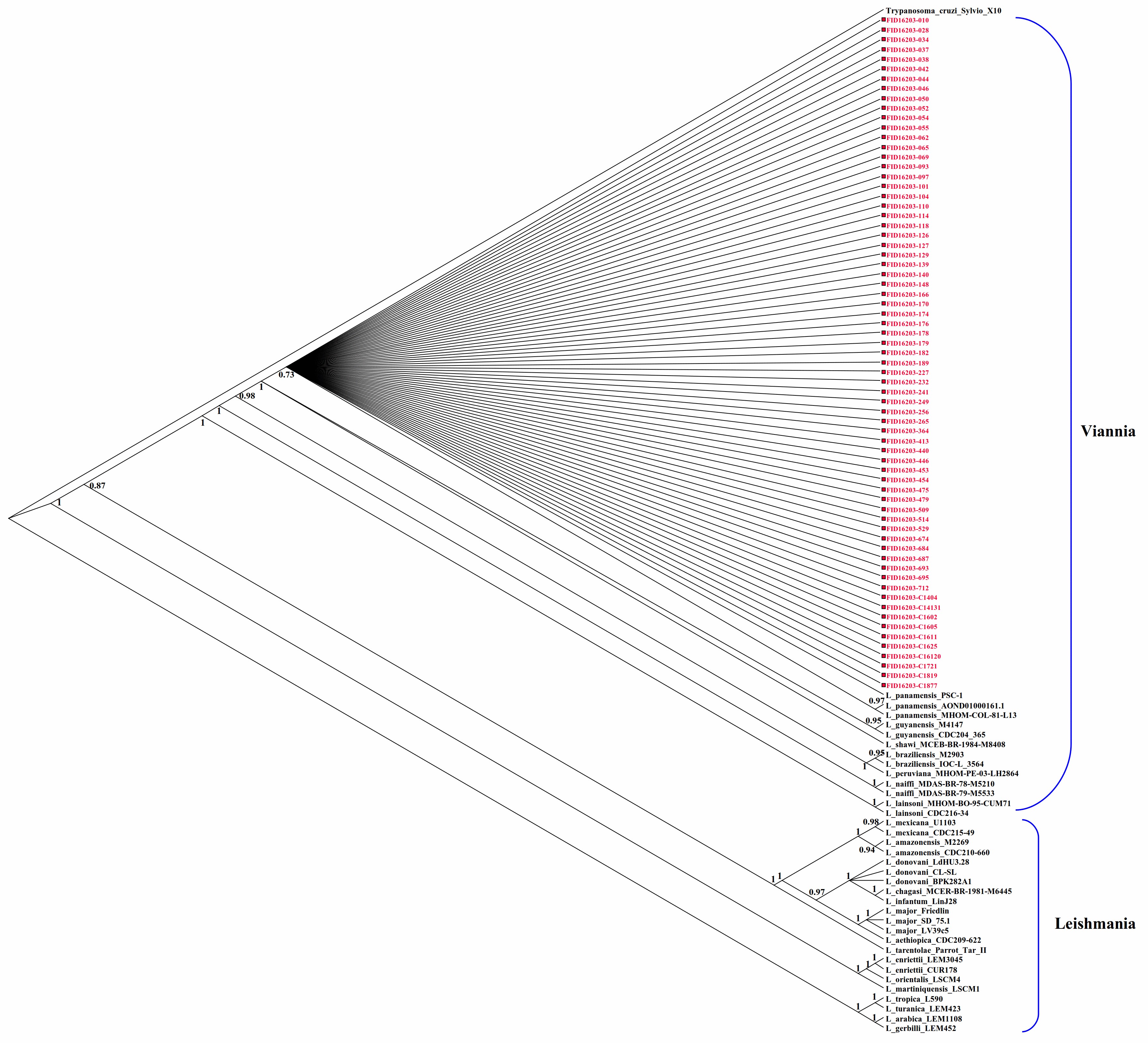

Supplement: Supplementary file 1 [file pathogens-12-00747-s001.zip › pathogens-2259471-supplementary/Supplementary Figure S1.jpg]
